# The HBx–CTTN interaction promotes cell proliferation and migration of hepatocellular carcinoma via CREB1

**DOI:** 10.1038/s41419-019-1650-x

**Published:** 2019-05-28

**Authors:** Yajun Li, Yongming Fu, Xingwang Hu, Lunquan Sun, Daolin Tang, Ning Li, Fang Peng, Xue-gong Fan

**Affiliations:** 1 Department of Infectious Diseases and Hunan Key Laboratory of Viral Hepatitis, Xiangya Hospital, Central South University, Changsha, China; 2 Center for Molecular Medicine, Xiangya Hospital, Central South University, Changsha, China; 3 Department of Surgery, UT Southwestern Medical Center, Dallas, Texas USA; 4 Department of Blood Transfusion, Xiangya Hospital, Central South University, Changsha, China; 5 NHC Key Laboratory of Cancer Proteomics, XiangYa Hospital, Central South University, Changsha, China

**Keywords:** Cancer, Viral hepatitis

## Abstract

Hepatitis B virus-encoded X protein (HBx) acts as a tumor promoter during hepatocellular carcinoma (HCC) development, probably by regulating the expression of host proteins through protein–protein interaction. A proteomics approach was used to identify HBx-interacting proteins involved in HBx-induced hepatocarcinogenesis. We validated the proteomics findings by co-immunoprecipitation and confocal microscopy. We performed cell proliferation, migration assays and cell cycle analyses in HCC cells. Finally, we confirmed the clinical significance of our findings in samples from patients. We found that cortactin (CTTN) is a novel HBx-interacting protein, and HBx regulates the expression of CTTN in the HCC cell lines MHCC-LM3 and HepG2. Mechanistically, by upregulating the expression of cAMP response element-binding protein (CREB1) and its downstream targets, such as cyclin D1 and MMP-9, the effects of the HBx-CTTN interaction on the enhancement of cellular proliferation and migration were maintained by inhibiting cell cycle arrest. In addition, we demonstrated that the levels of CTTN and CREB1 were closely correlated in clinical samples from HBV-infected patients with HCC. Overall, our data suggests that HBx contributes to cell migration and proliferation of HCC cells by interacting with CTTN and regulating the expression of CTTN and CREB1. Therefore, the HBx/CTTN/CREB1 axis is a potential novel therapeutic target in HCC.

## Introduction

Hepatocellular carcinoma (HCC) is an aggressive human malignancy^1–3^. Hepatitis B virus (HBV) infection is the most common risk factor for HCC, and there are ~250 million chronic HBV carriers in the world^4–7^. Although HBV vaccines have obviously decreased the number of new HBV infection cases, they have limited benefits for previously diagnosed patients with chronic HBV infection^8,9^. The HBV genome comprises four overlapping open-reading frames: preC/C, preS/S, P, and X^10^. The X protein (HBx), encoded by the *X* region, is a multifunctional viral regulator involved in viral pathogenesis and carcinogenesis, and it plays an important role in HBV-related HCC processes, such as autophagy regulation, DNA repair inhibition, post‐transcriptional regulation, and cell cycle arrest^9,11–14^.

HBx interacts with many host targets, and as such, it is important in viral hepatocarcinogenesis^15–19^. For example, HBx activates transcription by interacting with transcription factors or complexes, including P53, C/EBP, Sp1, and STAT3^20–27^. HBx also activates various cellular signal transduction pathways related to its transactivation, such as the NF-κB, AMP-activated protein kinase (AMPK), and MEKK1/Jun kinase signaling pathways^22,28^. Thus, the identification of novel HBx–host interactors and the pathways they are involved with might aid in the development of effective therapies for patients with HCC.

In this study, we looked for HBx-interacting proteins in HCC and explored their mechanism of action. Our data demonstrated the physical association between HBx and CTTN and identified the new HBx/CTTN/CREB1 axis as a crucial switch regulating the proliferation and migration of HCC cells.

## Methods

### Cell lines, cell culture, and cell transfection

The HCC cell lines HepG2 and MHCC-LM3 were obtained from the American Type Culture Collection (ATCC, Manassas, VA, USA) and China Center for Type Culture Collection (CCTCC, China) and cultured in Dulbecco’s Modified Eagle’s medium (DMEM) supplemented with 10% (v/v) fetal bovine serum (FBS) at a humidified incubator with 5% CO_2_ at 37 °C.

HBx and CREB1 overexpression was achieved by transfection of HBx (Plasmid #42596, Addgene, MA, USA) or CREB1-overexpressing vectors (GeneCopoecia, Guangzhou, China). The empty vector pcDNA3.1 (vector NC) was used as control; CTTN knockdown was achieved by transfection of small interfering RNAs targeting CTTN (si-CTTN; GeneCopoeia, Maryland, USA). HCC cells (1.5 × 10^5^) grown on six-well plates were transfected with 100 pmol siRNA or 2 µg of overexpression vectors using 6 µL of Lipofectamine 2000 (#11668019, Invitrogen, MA, USA) as described by the manufacturer. The cells were harvested after 48 h. Western blot analyses or other experiments were performed. The siRNA and vector construction primer sequences are listed in Table S1.

### Establishment of stably HBx-expressing HepG2 cells

Target HBx sequences of pcDNA3.1-Flag HBx plasmid (Plasmid #42596, Addgene, MA, USA) were amplified by PrimeSTAR GXL DNA polymerase (#R050A, Takara, Dalian, Japan). Next, HBx gene products and pLV-cDNA (No. 632177, Clontech, CA, USA) were amplified via enzyme digestion and purification according to the manufacturer’s instructions. After the enzyme-linked reaction, amplification, and DNA sequencing, we constructed a recombinant pLV-cDNA-HBx plasmid through the recombination of the digested HBx fragment and the purified pLV-cDNA product. According to the lentiviral packaging system protocol (Clontech, CA, USA), we collected lentiviral particles by transient transfection of 293 T cells, and then infected HepG2 cells with lentivirus. A Blasticidin S (#60218ES10, Yeasen, Shanghai, China) concentration of 6 µg/mL selected stably expressing *HBx* target cells. In this study, the stably modified cells (HBx-HepG2) were only used in the identification of proteins interacting with HBx, verification of the interaction between CTTN and HBx, and determination of CTTN mRNA levels and CTTN protein stability experiments.

### Immunoprecipitations

Stably HBx-expressing HepG2 cells were lysed in a co-immunoprecipitation lysis buffer (buffer composed of 20 mM Tris (pH 7.5), 150 mM NaCl, and 1% Triton X-100; P0013, BBI, Shanghai, China) containing a protease inhibitor cocktail for 60 min at 4 °C. After centrifugation, the supernatant was incubated with anti-mouse IgG (#3420, CST, MA, USA) at 4 °C for 60 min and then with 20 μL of protein G agarose beads (#37478, CST, MA, USA); an anti-HBx antibody (M10514, Xiamen Innovax Biotech, Xiamen, China) was then added, and the mixture was incubated at 4 °C overnight. Protein complexes containing the HBx antibody were precipitated with anti-mouse IgG beads, and washed with RIPA lysis buffer (P0013, BBI, Shanghai, China) and phosphate-buffered saline (PBS), sequentially. Finally, the pellet was eluted and subjected to SDS-PAGE analysis.

### Mass spectrometry (MS) and database analysis

LC-ESI-LTQ-Orbitrap-MS analysis of proteins was performed as previously described by us^29^. The protein bands were excised and destained with 100 mM NH_4_HCO_3_ in 50% acetonitrile (ACN). The proteins were reduced (10 mM dithiothreitol, 56 °C, 30 min), alkylated (50 mM iodoacetamide, in the dark, 20−25 °C, 30 min), and dried in a vacuum centrifuge. The gel pieces that contained proteins were incubated in a digestion solution (40 mM NH_4_HCO_3_, 9% ACN, and 20 μg/mL trypsin) at 37 °C for 18–24 h. The tryptic peptide mixture was purified with a ZipTipC18 microcolumn (cat. no. ZTC18S096; Millipore, Darmstadt, Germany). The purified tryptic peptide mixture was separated onto a PepMap C18 trap column (75 μm, 15 cm) at a column flow rate of 200 nL/min. The MS scan and MS spectra were measured in data-dependent mode with MS/MS analysis of the seven strongest ions in the LTQ. The MS data were analyzed using the Xcalibur software and submitted to the database search via Proteome Discoverer. Search parameters included taxonomy (*Homo sapiens*), enzyme (trypsin), MS/MS tolerance (±0.5 Da), peptide tolerance (20 ppm), peptide charge (2 + , 3 + , and 4 + ), missed cleavage sites (2), fixed modification (carbamoyl methylation of cysteine), and variable modification (methionine oxidation).

### Western blot analysis

Cells were lysed in RIPA lysis buffer (BBI) and separated by electrophoresis, transferred to membranes, and subjected to western blot according to the standard procedure. The primary antibodies used included anti-HBx (dilution 1:1000, M10514, Xiamen Innovax Biotech, Xiamen, China), anti-CTTN (dilution 1:1000, ab81208, Abcam), anti-CREB1 (dilution 1:1000, Cat. #9197, CST), anti-cyclin D1 (dilution 1:1000, D220509, BBI), anti-MMP-9 (dilution 1:1000, ab73734, Abcam), anti-GAPDH (dilution 1:2000, ab8245, Abcam), anti-E-cadherin (Cat. 20874, Proteintech), and anti-vimentin (dilution 1:1000, sc-80975, Santa Cruz). After incubating with goat anti-mouse and goat anti-rabbit secondary antibodies for 1 h at room temperature, immunoreactive bands were visualized with a chemiluminescence system and quantified using Image J software.

### Immunofluorescence staining

For the detection of the intracellular distribution of HBx and CTTN, cells (1 × 10^5^ per well) were seeded in six-well glass-bottom plates, fixed in 4% paraformaldehyde for 15 min, and then permeabilized with 0.2% Triton X-100 (PBS) for 10 min. Nonspecific binding sites were blocked with 1% bovine serum albumin in PBS for 1 h. Cells were treated with a primary antibody specific for HBx (1:1000, M10514, Xiamen Innovax Biotech) or CTTN (dilution 1:1000, ab81208, Abcam) overnight at 4 °C. Thereafter, the cells were incubated with eFluor570-F(ab’)2-goat anti-mouse IgG/IgM (H + L) (dilution 1:20, Cat. # 41-4010-82, Invitrogen) and FITC-goat anti-Rabbit IgG (H + L) cross-adsorbed secondary antibody (dilution 1:20, Cat. #F-2765, Invitrogen, Carlsbad, CA, USA). DAPI (Beyotime, Shanghai, China) was used to stain nuclei before capturing images. The images were acquired using a confocal microscope (Zeiss, Oberkochen, Germany). The green fluorescence indicated HBx expression, the red fluorescence indicated CTTN expression, and the blue fluorescence indicated the nuclei.

### Protein–protein interaction analysis

The Mentha (http://mentha.uniroma2.it/about.php), BioGrid (https://thebiogrid.org/), IntAct (https://www.ebi.ac.uk/intact/), and BIND (https://www.bindingdb.org/bind/aboutus.jsp) databases were used to analyze the proteins interacting with HBx or CTTN. The candidate proteins were then applicated for Kyoto Encyclopedia of Genes and Genomes (KEGG) pathway annotation (http://www.genome.jp/kegg/) to identify the related pathways. The interaction network of these proteins was constructed using String (https://string-db.org/) and visualized with the Cytoscape software (version 3.4) 18.

### MTT assays

MTT assays were performed to evaluate cell viability. Twenty-four hours after seeding into 96-well plates (5 × 10^3^ cells/well), HepG2 and MHCC-LM3 cells were transfected with si-CTTN; 48 h after transfection, 20 μL of MTT (at a concentration of 5 mg/mL; Sigma-Aldrich) was added, and the cells were incubated for an additional 4 h in a humidified incubator with 5% CO_2_ at 37 °C. DMSO (200 μL) was added after the supernatant was discarded to dissolve the formazan crystals, and finally absorbance at 490 nm was measured (PerkinElmer). The viability of non-treated cells (control) was defined as 100%, and the viability of the other groups of cells was calculated accordingly.

### 5-Bromo-2-deoxyuridine assays

5-Bromo-2-deoxyuridine (BrdU) assays were conducted 24 and 48 h after cells were co-transfected with si-CTTN and pcDNA3.1( + )-HBx-overexpressing vectors. Cells were seeded in 96-well culture plates at a density of 2 × 10^3^ cells/well, cultured for 48 h, and then incubated with a final concentration of 10 μM BrdU (BD Pharmingen, San Diego, CA, USA) for 2 h. Cells were fixed for 30 min at room temperature, incubated with peroxidase-coupled BrdU antibody (Sigma-Aldrich) for 60 min at room temperature, washed three times with PBS, incubated with peroxidase substrate (tetramethylbenzidine) for 30 min, and the 450-nm absorbance values were measured for each well. Background BrdU immunofluorescence was determined in cells not exposed to BrdU and stained with the BrdU antibody.

### Transwell assays

Cells were co-transfected with si-CTTN and pcDNA3.1( + )-HBx-overexpressing vectors, and seeded on the top side of polycarbonate transwell filters. The cells were suspended in a medium without serum, while the bottom chamber was filled with medium with serum. After 24 h, the non-migrating cells in the top chambers were removed with cotton swabs. The migrated cells on the lower membrane surface were fixed in 100% methanol for 10 min, air-dried, stained with crystal violet (BBI, Shanghai, China), and counted under a microscope.

### Cell cycle analysis

Cells (5 × 10^5^) were silenced for *CTTN* or transfected with a plasmid CREB1 knockdown for 48 h. The cells were washed with PBS and collected into a 1.5-mL centrifuge tube. Subsequently, the cells were fixed with 70% ethanol for 24 h and then incubated with a propidium iodide solution (C1052, BBI, Shanghai, China). Flow cytometry was used to study the cell cycle distribution after HBx overexpression and CREB1 knockdown, according to the manufacturer’s instructions (Millipore Guava easyCyte, ON, Canada).

### Quantitative PCR (qPCR) and tissue specimens

The total RNA was isolated from stably HBx-expressing HepG2 cells and HBx ( + )/(−) HCC tissues by using TRIzol (Invitrogen) following the manufacturer’s protocol. A SYBR green PCR Master Mix (Qiagen) was used for mRNA expression detection following the protocol of the manufacturer. The GAPDH expression was used as an endogenous control. The 2^−ΔΔCT^ method^30^ was used to calculate the relative fold changes.

Thirty HBx ( + ) and 22 HBx (−) HCC tissues were collected from tumor surgical resection in the Xiangya Hospital (Central South University, Changsha, China) with the approval of the Medical Ethics Committee of Xiangya hospital at Central South University. Informed consent was obtained from all patients enrolled.

### Statistical analysis

The data were processed using SPSS 17.0 statistical software and presented as the mean ± SD of at least three independent experiments. A Student's *t* test was used for statistical comparison between means, where applicable. Differences between more than two groups were estimated using one-way ANOVA. *P < 0.05; **P < 0.01.

## Results

### Identification of proteins interacting with HBx in HepG2 cells

**Fig. 1:**
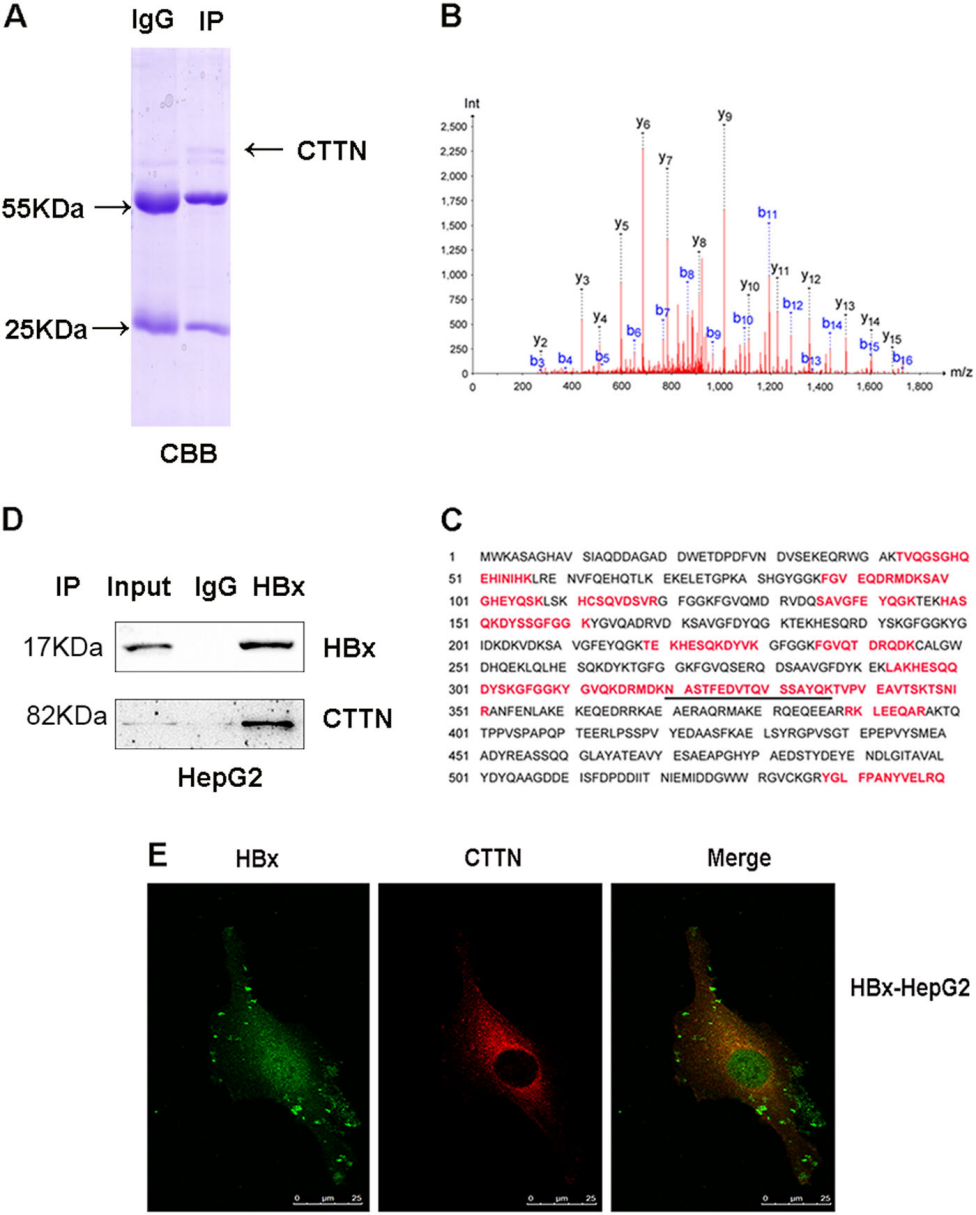
CTTN interacts with HBx in HepG2 HCC cells. **a** Analysis of HBx-associated proteins by CoIP-MS in HepG2 cells overexpressing HBx (HBx-HepG2). **b** Representative MS/MS spectrum of CTTN; the amino acid sequence of a doubly charged peptide with m/z 937.9402 was identified as NASTFEDVTQVSSAYQK from mass differences in the y and b fragment ions series. **c** Protein sequence of CTTN: the matched peptides are labeled in red bold letters. **d** CoIP to validate the HBx-CTTN interaction. CTTN was immunoprecipitated by an anti-HBx but not by IgG control. **e** Expression of HBx and CTTN in the cytoplasm HBx-HepG2 by confocal microscopy

To identify proteins that interact with HBx, we used a CoIP-MS proteomic analysis of the HBx complexes isolated from HepG2 stably expressing HBx (HBx-HepG2) cells using a HBx antibody; nonimmune IgGs were used as a negative control. The complexes were eluted and separated on 12% SDS-PAGE (Fig. 1a). Proteins in the gels were detected by Coomassie brilliant blue staining (CBB), in-gel trypsin digested, and subjected to MS analysis. Fifteen proteins were identified in the anti-HBx-treated cells and not in the negative control: their mass spectra are shown as supplementary data (Table S2). Among them, CTTN was identified from a specific band that originated the highest score and 18 unique peptides. We confirmed the protein sequence of CTTN (Fig. 1b–c; the matched peptides are labeled in red bold letters).

### Verification of the interaction between CTTN and HBx

We confirmed the interaction between CTTN and HBx in co-immunoprecipitation assays. HBx-HepG2 cell lysates were immunoprecipitated with an HBx antibody or IgG; immunocomplexes were separated on a gel and subjected to western blot with a CTTN antibody. As shown in Fig. 1d, CTTN was detected in anti-HBx immunoprecipitates but not in the control samples, suggesting the interaction between CTTN and HBx in vitro. Subsequently, we investigated CTTN and HBx subcellular localization in HBx-HepG2 cells to determine where they interacted with each other. Immunofluorescence staining indicated that HBx enhanced CTTN fluorescence intensity (Fig. 1d; Supplementary Fig. S1). Moreover, CTTN and HBx colocalized in the cytoplasm (Fig. 1e).

### Roles of HBx/CTTN interaction in enhancing cell proliferation and migration

**Fig. 2:**
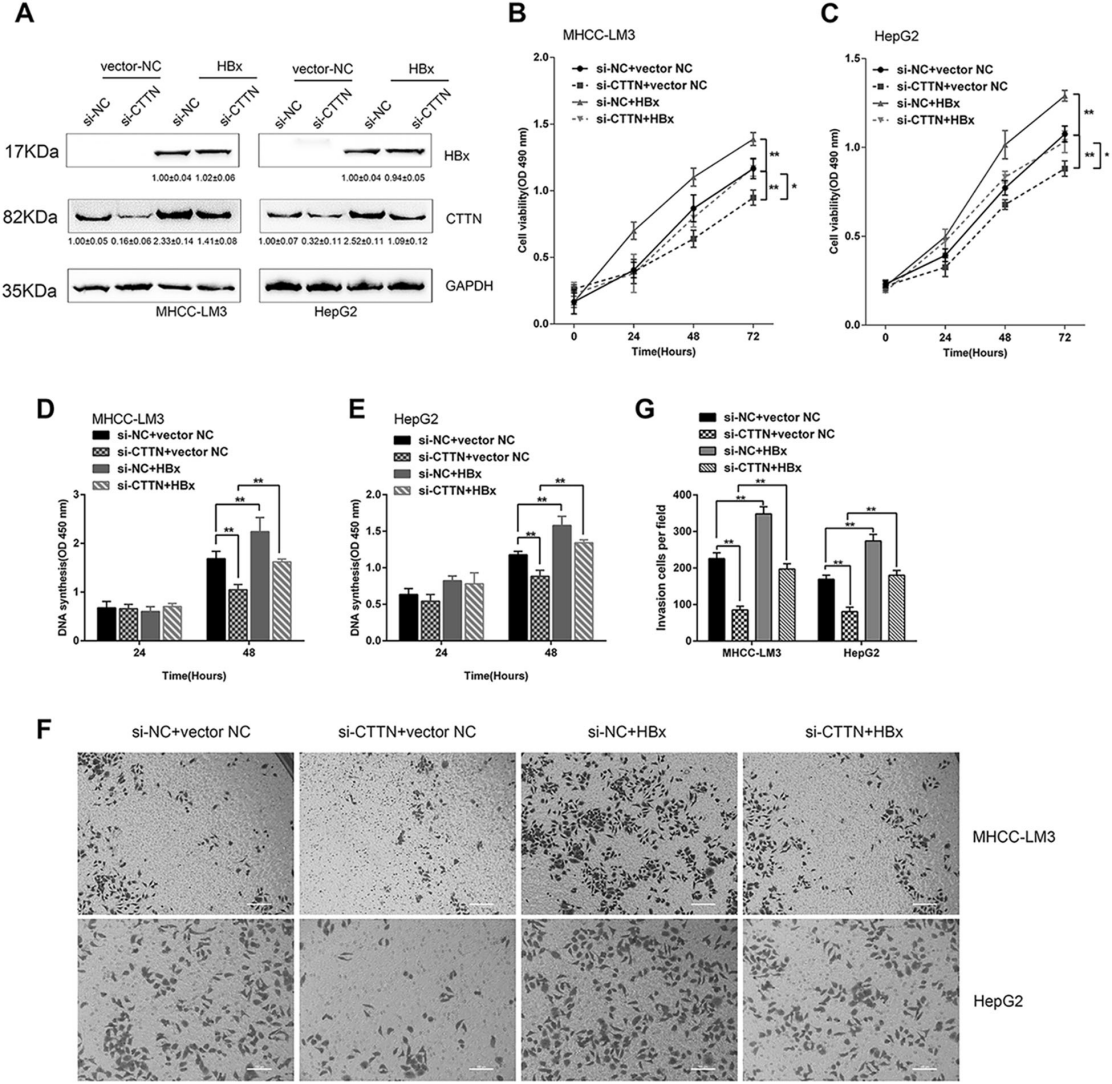
Effects of the HBx-CTTN interaction on cell proliferation and migration. **a** HepG2 and MHCC-LM3 cells were co-transfected with si-CTTN and pcDNA3.1( + )-HBx-overexpressing vectors. Protein expressions of HBx and CTTN were measured by western blot. **b**, **c** HepG2 and MHCC-LM3 cells were co-transfected with si-CTTN and pcDNA3.1( + )-HBx-overexpressing vectors and the cell viability was examined using MTT assays. **d**, **e** DNA synthesis was examined in BrdU assays; the data are presented as the mean ± SD of three independent experiments. **f**, **g** Migration of the indicated groups of cells examined using transwell assays on a light microscope. The statistical analysis was shown in the right panel. *P < 0.05, **P < 0.01

To investigate whether the HBx/CTTN interaction had an impact on cellular processes, we conducted MTT, BrdU, and transwell assays. We found that HBx significantly increased the CTTN in HepG2 and MHCC-LM3 cells. While *CTTN* silencing could partially reverse the effect of HBx on CTTN protein expression (Fig. 2a). The upregulation of CTTN expression by HBx might be associated with the increase of protein stability (Supplementary Fig. S2). MTT and BrdU assays showed that the viability and DNA synthesis ability of HepG2 and MHCC-LM3 cells increased upon HBx overexpression and decreased upon *CTTN* silencing, and *CTTN* silencing reversed the effects of HBx overexpression in cells that overexpressed HBx and were silenced for *CTTN* (Fig. 2b–e). Similarly, transwell assays revealed that cell migration was significantly suppressed by *CTTN* knockdown and promoted by HBx overexpression (Fig. 2f, g). These findings indicate that HBx promotes cell proliferation and migration of HCC cells by interacting with CTTN.

### HBx-CTTN interaction modulates CREB1 and its downstream target genes

**Fig. 3:**
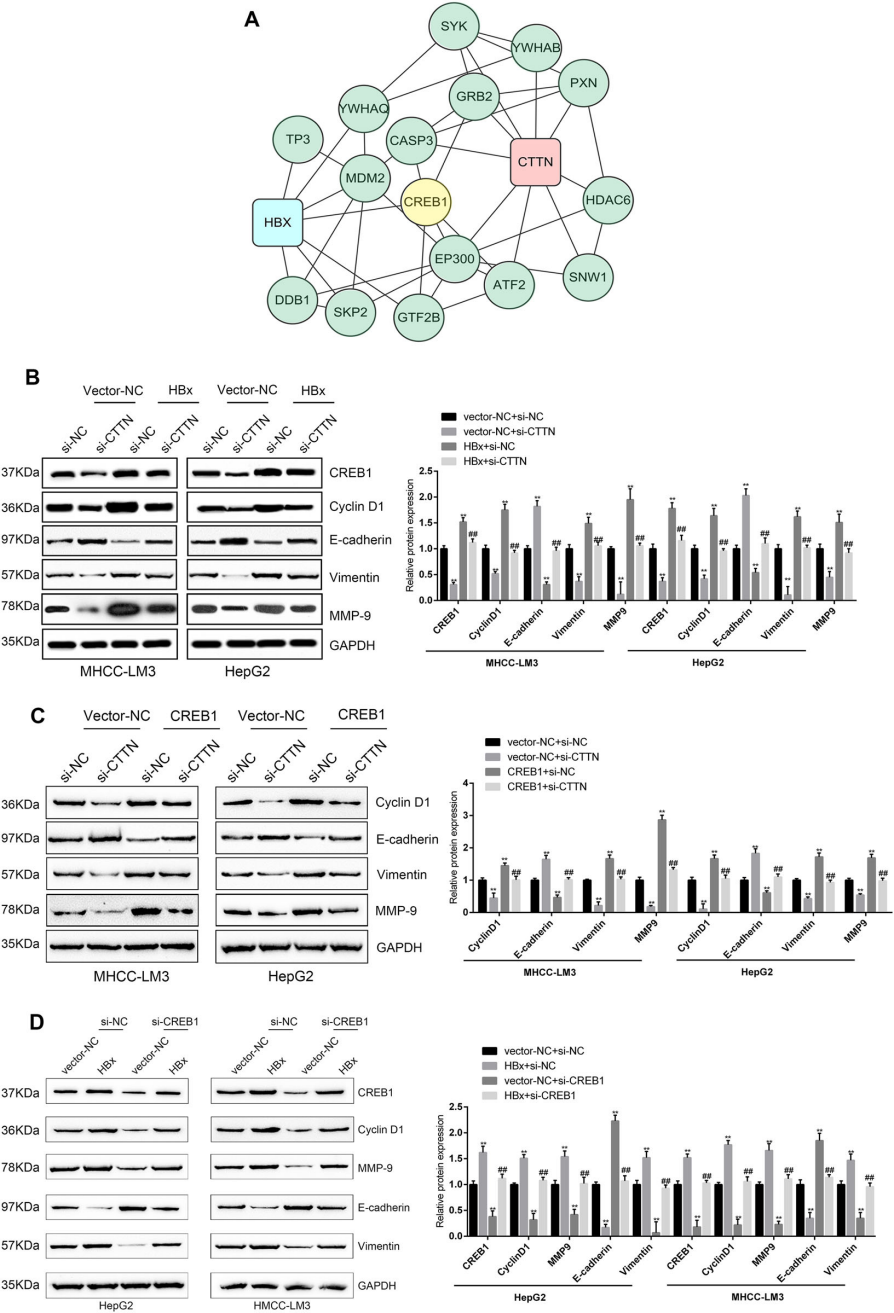
The HBx-CTTN interaction modulates CREB1 and its downstream target genes. **a** Analysis of HBx and CTTN protein–protein network using online databases. The network nodes represent proteins, and the connecting lines represent protein–protein interactions. **b** CREB1, cyclin D1, MMP-9, E-cadherin, and vimentin were detected by western blot in HepG2 and MHCC-LM3 cells. HepG2 and MHCC-LM3 cells were transfected with si-CTTN or si-NC and pcDNA3.1( + )-HBx or vector NC. si-CTTN or si-NC and pcDNA3.1( + )-HBx or vector NC were compared with the si-NC + vector-NC group. **P < 0.01. si-CTTN and pcDNA3.1( + )-HBx group were compared with HBx + si-NC group. ^##^*P* < 0.01. **c** The expression of EMT-associated proteins was detected in HepG2 and MHCC-LM3 cells transfected with si-CTTN or si-NC and CREB1 or vector NC. si-CTTN or si-NC and CREB1 or vector NC were compared with the si-NC + vector-NC group. **P < 0.01. si-CTTN and the CREB1 group were compared with the CREB1 + si-NC group. ^##^*P* < 0.01. **d** The expression of CTTN, HBx, CREB1, cyclin D1, MMP-9, E-cadherin, and vimentin was determined by western blot when HepG2 and MHCC-LM3 cells were transfected with si-CREB1 or si-NC and pcDNA3.1( + )-HBx or vector NC compared with the si-NC + vector-NC group. **P < 0.01. si-CREB1 and pcDNA3.1( + )-HBx group were compared with the HBx + si-NC group. ^##^*P* < 0.01

To investigate how HBx-CTTN modulates cell proliferation and migration, we performed bioinformatics analyses and analyzed the signaling pathways that are involved (Fig. S3). Proteins that might be related to HBx and CTTN were analyzed using online databases. A total of 124 human proteins were predicted to interact with CTTN (Table S3), and a total of 20 proteins were predicted to bind to HBx (Table S4). KEGG pathway annotation (Tables S5 and S6) showed that 16 proteins (nine for CTTN and seven for HBx) were involved in viral carcinogenesis (hsa05023); these proteins were selected and visualized using Cytoscape. We found that, among them (Fig. 3a), a cellular transcription factor was reported to participate in HBx-induced dysregulation of lipogenesis in HCC cells^31^. Previous studies have shown that *CREB1* knockdown inhibits the expression of its downstream targets: cyclin D1, Bcl-2, and MMP-9 in gastric cancer^2^. Based on its essential role in HCC, CREB1 was chosen as a potential target. Therefore, we decided to focus on CREB1 as the possible target of HBx-CTTN.

**Fig. 4:**
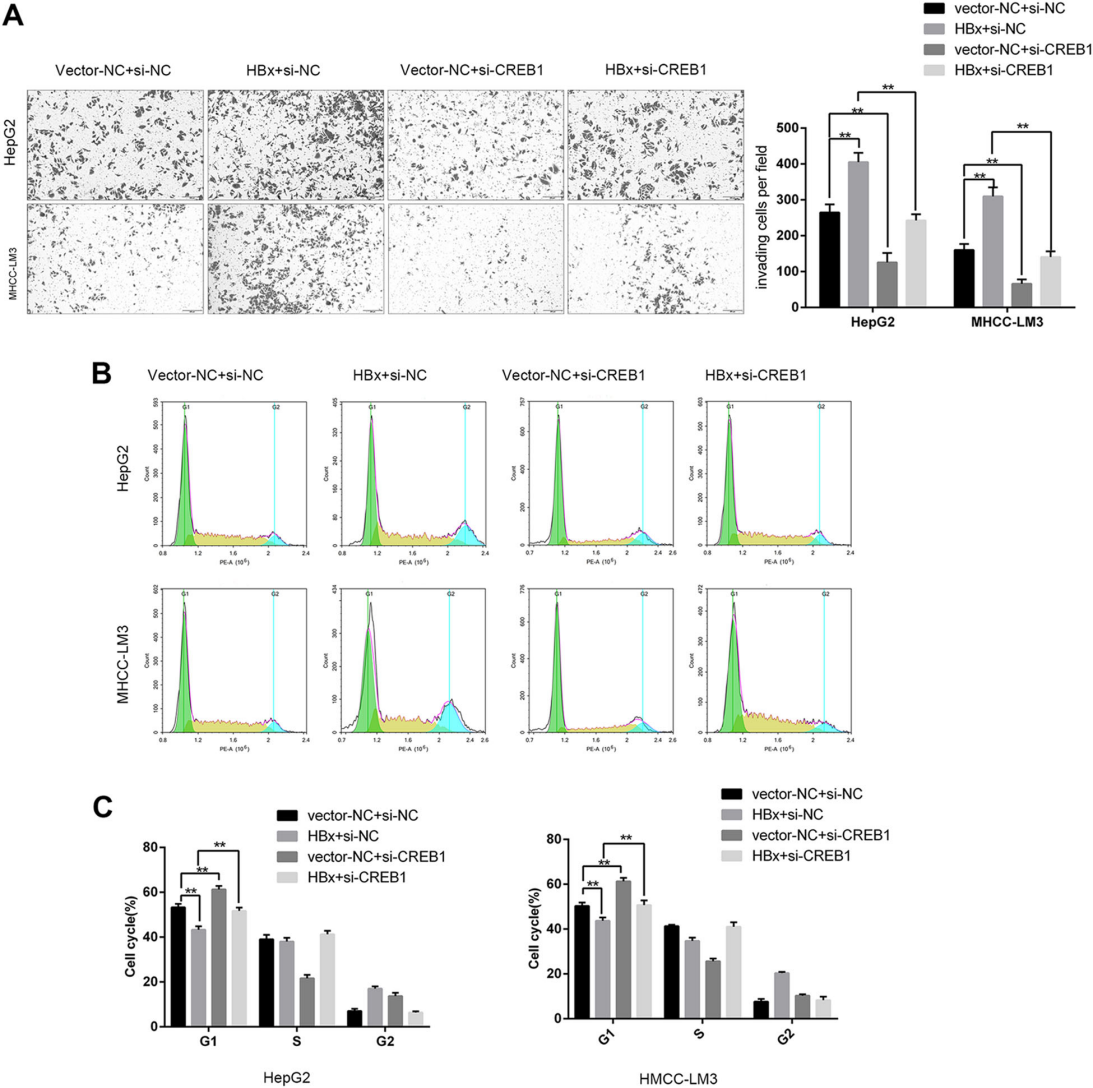
HBx-CTTN regulates cell proliferation and migration via CREB1. HepG2 and MHCC-LM3 cells were transfected with si-CREB1 or si-NC and pcDNA3.1( + )-HBx or vector NC. **a** Migration of the indicated groups of cells was examined using transwell assays. **b**, **c** Cell cycle analysis by flow cytometry in HepG2 and MHCC-LM3 cells. The results show the percentage of cells in each phase of the cell cycle. *P < 0.05, **P < 0.01

To evaluate the combined effects of HBx and CTTN on CREB1 and its downstream target genes, MHCC-LM3 and HepG2 cells were silenced for CTTN, and the protein levels of CREB1, cyclin D1, E-cadherin, vimentin, and MMP-9 were examined. We found that CTTN knockdown remarkably increased E-cadherin protein levels and reduced the levels of CREB1, cyclin D1, vimentin, and MMP-9, while HBx overexpression decreased E-cadherin expression and increased the levels of CREB1, cyclin D1, vimentin, and MMP-9, and the effect of HBx overexpression was partially attenuated by CTTN knockdown (Fig. 3b). The epithelial–mesenchymal transition (EMT) is directly linked to tumor initiation and invasion^32^. Therefore, we monitored the expression of EMT markers and found that CREB1 overexpression significantly reduced E-cadherin expression and increased vimentin and MMP-9 levels in MHCC-LM3 and HepG2 cells, and this effect was reversed by CTTN knockdown (Fig. 3c). We further assessed the relationship between HBx-CTTN and CREB1. The western blot results showed that CREB1 knockdown increased E-cadherin expression and reduced MMP-9 and vimentin expression;these effects were reversed by HBx overexpression (Fig. 3d). We found that CREB1 knockdown inhibited cell migration and induced cell cycle arrest at G1, while these effects could be reversed by HBx overexpression (Fig. 4a–c). Therefore, our results suggested that CREB1 and its downstream targets can be regulated by the HBx-CTTN complex.

### Positive correlation between the high expression of CTTN and CREB1 in HBV-associated patients

**Fig. 5:**
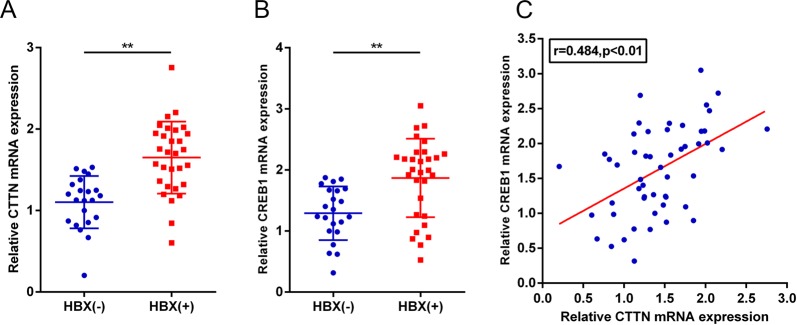
Upregulation of CTTN and CREB1 in clinical specimens. **a**, **b** *CTTN* and *CREB1* expression in 30 HBx ( + ) and 22 HBx (−) HCC tissues from patients, as assessed by qPCR. The data are presented as mean ± SD of three independent experiments. **P < 0.01. **c** The correlation between *CTTN* and *CREB1* expression in tissue samples was analyzed using Spearman’s rank correlation analysis

To investigate the clinical significance of our findings, the mRNA levels of *CTTN* and *CREB1* were evaluated by qPCR in HCC tissues of patients. As shown in Fig. 5, the levels of *CTTN* in the HBx ( + ) group were significantly upregulated compared with the HBx(−) group (*P* < 0.01), and similar results were obtained with *CREB1* (*P* < 0.01). In addition, *CTTN* and *CREB1* expression were positively correlated in tissue samples.

## Discussion

HBx viral protein contributes to the development of HCC mainly through HBx–host interactions, and it is involved in numerous cellular activities, including transcription activation, transactivation, DNA damage repair, and cell transformation inhibition^7,9,13,23,33^. For example, HBx inhibits DNA repair by interacting with PARP1 and SIRT6, resulting in dissociation of SIRT6 and PPAP1^9^. HBx also inhibits cell apoptosis by regulating the endoplasmic reticulum (ER) stress response: GRP78 interacts with HBx in the ER, and this interaction leads to the interruption of ER stress response and host cell apoptosis^17^. Many studies have shown that HBx participates in the regulation of viral or host proteins by protein–protein interactions, providing potent evidence for HBV-related tumorigenesis. However, how HBx protein induces HCC is still unclarified. In this study, we undertook a proteomics approach to identify HBx-interacting proteins and found that CTTN, a Src substrate, was a novel HBx-interacting protein.

Previous studies have shown that Src protein and RNA expression levels are significantly upregulated in most HBV^+^–HCC specimens and *HBx* transgenic mice, and the overexpression of Src promotes cell viability, migration, and cell colony formation^34–36^. The Src substrate CTTN has been found to be overexpressed in various cancers, including human colorectal cancer, esophageal tumors, and non-small-cell lung cancer, and HCC and CTTN overexpression closely correlates with tumor aggressiveness, cell adhesion, and cell motility^37–42^. We demonstrated that HBx contributed to cell migration and proliferation of HCC by binding CTTN in the cytoplasm and upregulating its expression. It has been reported that the phosphorylation and activation of Src are increased in HCC and result in the phosphorylation of CTTN at Y421^36,43,44^. This modification is closely associated with CTTN localization and function through the regulation of its interaction with other host proteins^45^. We believe that the adjacent region between Y421 and the SH3 domain of CTTN (aa 490–550) in the C terminus contributes to the phosphorylation of CTTN at Y421 upon HBx-CTTN interaction, and affects the structure and biological functions of CTTN. This hypothesis needs further investigation. Furthermore, we found that HBx regulates CTTN protein expression at the post-transcriptional level, and HBx increases CTTN protein stability (Figure S2). Given that Zhao J. et al. reported that CTTN protein degradation was conducted by ubiquitin-independent proteasome system, we assumed that HBx could increase the stability of CTTN through attenuation of CTTN’s ubiquitination^46^.

We also performed bioinformatics analyses to identify the potential targets of HBx and CTTN. Based on its essential role in HCC, CREB1 was chosen as a potential target. We hypothesized that the HBx-CTTN interaction may affect CREB1 and its downstream targets. CREB1 is part of leucine zipper family of proteins, and its expression is essential for various cell functions, including apoptosis, cell cycle, and DNA repair^47–49^. Once activated, CREB1 regulates its downstream target genes, including the apoptosis suppressor gene *Bcl2*, a metalloproteinase involved in extracellular matrix remodeling, *MMP9*, and the cell cycle-related genes, cyclin D1, A1, and B1^2,50^. Previous studies have revealed that HBx can induce CREB-mediated activation of *miR-3188* and Notch signaling, resulting in the development of HBV-related HCC^51–53^. Based on our data, we illustrate a critical oncogenic role of CREB1, which is overexpressed and correlates with CTTN in HBV^+^ HCC. Furthermore, by targeting CREB1, the HBx-CTTN interaction forms a critical oncogenic axis, which regulates cell proliferation, migration, and oncogenic signaling in HCC. Bioinformatics analysis has shown that SKP2, a vital component of the SCF ubiquitin E3 ligase, is involved in HBV-related tumorigenesis^54^. CTTN also plays a role in SKP2 expression^55,56^. Therefore, the HBx-CTTN interaction has a possible effect on SKP2. This hypothesis might be investigated in the future.

Based on this work, we provided a novel mechanism of how HBx contributes to the progression of HCC: HBx interacts with CTTN in the cytoplasm, resulting in the upregulation of CREB1, which promotes the proliferation and migration of HCC. The crosstalk between CTTN and HBx may also provide a potential therapeutic strategy for HCC therapy.

## Supplementary information

Supplementary information

Table S1–6
