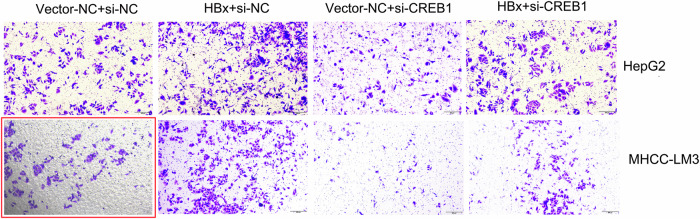# Correction: The HBx-CTTN interaction promotes cell proliferation and migration of hepatocellular carcinoma via CREB1

**DOI:** 10.1038/s41419-026-08854-3

**Published:** 2026-05-19

**Authors:** Yajun Li, Yongming Fu, Xingwang Hu, Lunquan Sun, Daolin Tang, Ning Li, Fang Peng, Xue-gong Fan

**Affiliations:** 1 Department of Infectious Diseases and Hunan Key Laboratory of Viral Hepatitis, Xiangya Hospital, Central South University, Changsha, China; 2 Center for Molecular Medicine, Xiangya Hospital, Central South University, Changsha, China; 3 Department of Surgery, UT Southwestern Medical Center, Dallas, TX USA; 4 Department of Blood Transfusion, Xiangya Hospital, Central South University, Changsha, China; 5 NHC Key Laboratory of Cancer Proteomics, XiangYa Hospital, Central South University, Changsha, China

Correction to: *Cell Death & Disease* 10.1038/s41419-019-1650-x, published online 28 May 2019

In the previously published paper, representative images of MHCC-LM3 transwell migration assay in Figure 2F and Figure 4A were incorrect. The corrected figures are provided below (shown in red frame), the text and figure legends remain the same. The correction does not affect the conclusions of this study. The authors regret these errors.

**Figure 2F-Published version** 
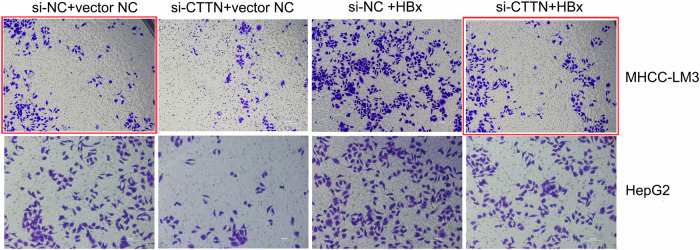


**Figure 2F-Corrected version** 
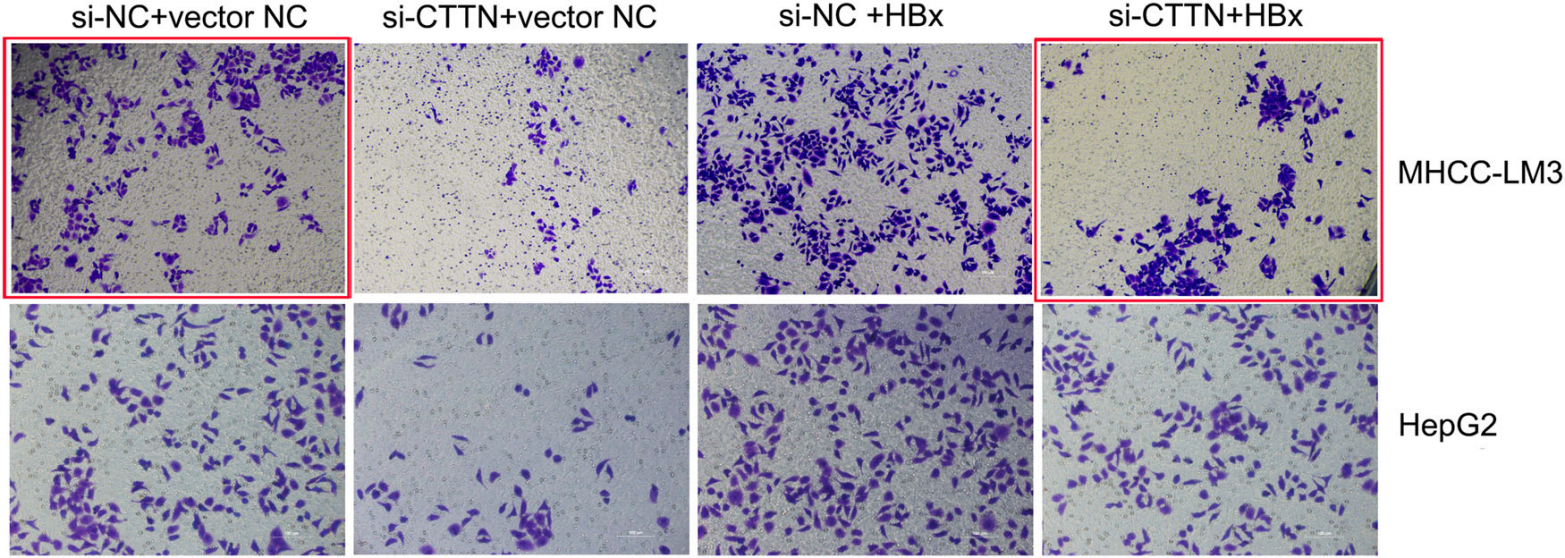


**Figure 4A-Published version** 
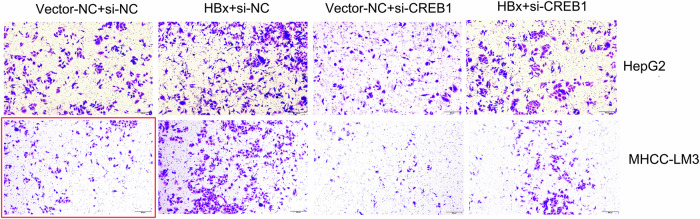


**Figure 4A-Corrected version**