# Supplementary material for: The HBx–CTTN interaction promotes cell proliferation and migration of hepatocellular carcinoma via CREB1
Source: Cell Death Dis. 2019 May 28;10(6):405. doi: 10.1038/s41419-019-1650-x (PMC6538608; doi:10.1038/s41419-019-1650-x)

Fig.S1 Expressions of HBx and CTTN in Immunofluorescence staining

Expressions of HBx and CTTN were visualized in the cytoplasm in HepG2 and MHCCLM3 cells by transfection of HBx or CTTN overexpressing vectors by immunofluorescence staining. Fluorescence images of HBx(green), CTTN(red), and merged images with DAPI(blue) are shown, respectively. Scale bar=100μm.

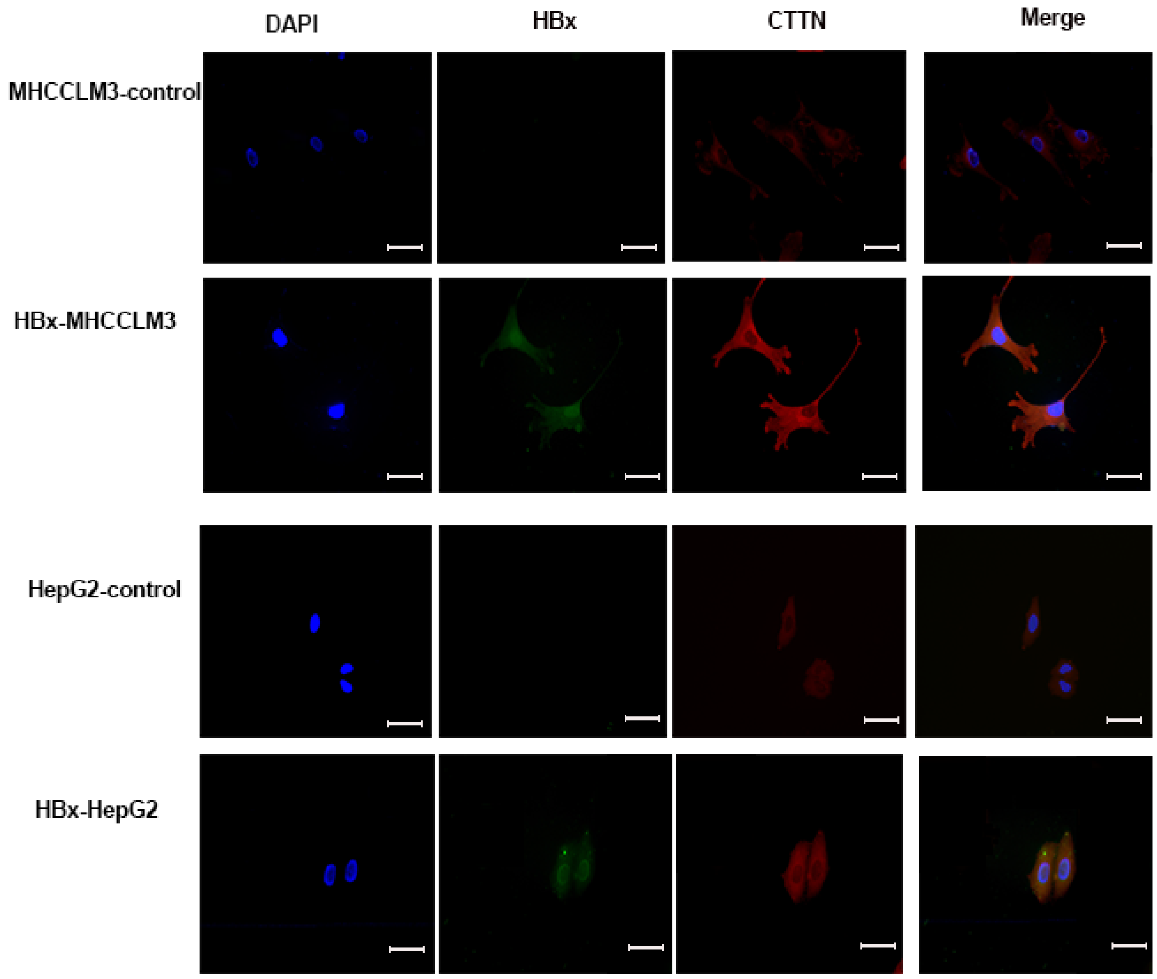

Fig.S2 HBx promoted protein stability of CTTN (A)The CTTN mRNA level of HepG2 and HBx overexpressing HepG2 cells was determined by QPCR. (B)HepG2 or HBx overexpressing HepG2 cells were exposed to 40 μg/mL CHX for indicated times (0, 4, 8, 12 hours) and CTTN protein level was examined by western blot.(C)Densitometry results of the CTTN immunoblots were plotted for half-life analysis using PRISM software.

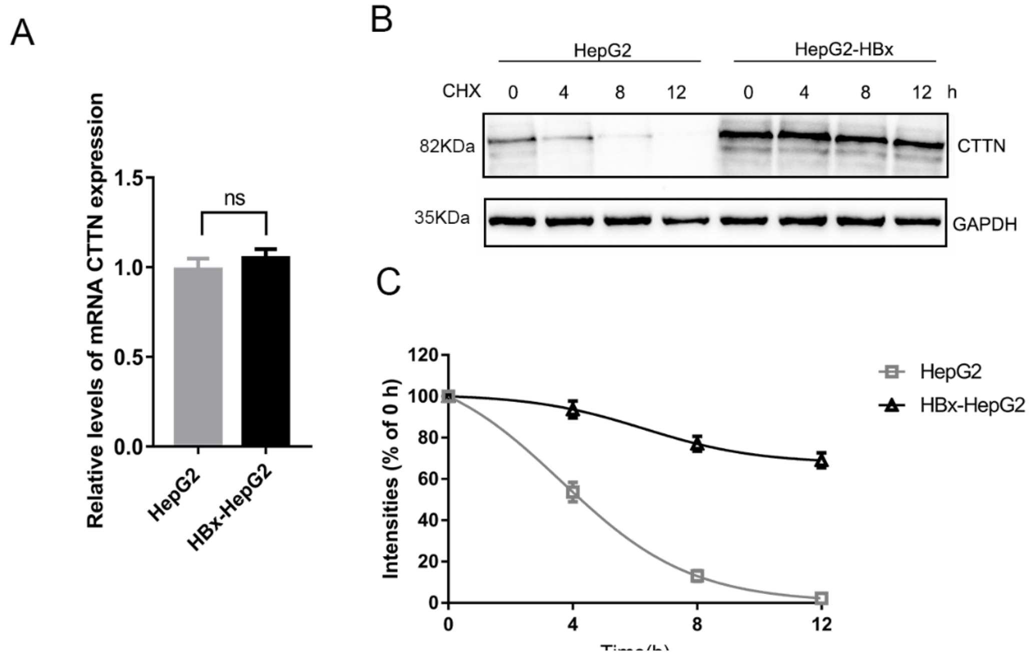

Fig.S3 The protein-protein interaction (PPI) network of CTTN and HBx in viral carcinogenesis Proteins that might interact with HBx or CTTN were analyzed using online databases, including Mentha, BioGrid, IntAct and BIND database. A total of 124 human proteins interacting with CTTN and 19 binding proteins of HBx were obtained. After KEGG pathway annotation, 16 specific proteins (9 for CTTN and 7 for HBx) involved in viral carcinogenesis (hsa05023) were selected for further analysis. The interaction network of these proteins was constructed from String (https://string-db.org/) and visualized

using Cytoscape software (version 3.4).

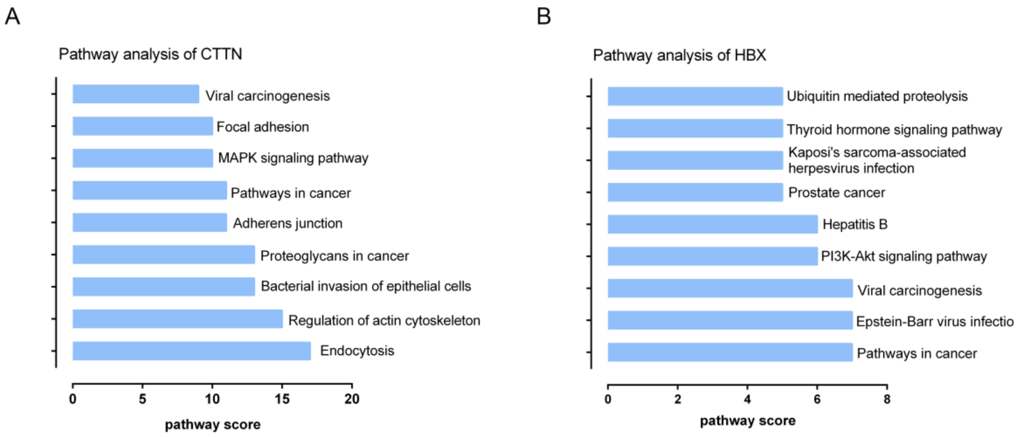

Supplement: Supplementary file 1 — Supplementary information [file 41419_2019_1650_MOESM1_ESM.pdf]
