# Supplementary material for: The HBx–CTTN interaction promotes cell proliferation and migration of hepatocellular carcinoma via CREB1
Source: Cell Death Dis. 2019 May 28;10(6):405. doi: 10.1038/s41419-019-1650-x (PMC6538608; doi:10.1038/s41419-019-1650-x)
Supplement: Supplementary file 2 — Table S1–6 [file 41419_2019_1650_MOESM2_ESM.docx]

**Table S1 Primer sequence**

| Name | Forward | Reverse |
| --- | --- | --- |
| HBx overexpression vector | CCGCTCGAGATGGCTGCTAGGCTGTGCT | CCGGAATTCTTAGGCAGAGGTGAAAAAGTTG |
| CREB1  overexpression vector | CCGCTCGAGATGACCATGGAATCTGGAGCC | CCGGAATTCTTACAATTTTTGAGTACCTTGAACA |
| Si-CTTN | GCAACUUAUUGUAUCUGAATT | UUCAGAUACAAUAAGUUGCTT |
| Si-CREB1 | CAAACAGUUCAGAUUUCAATT | UUGAAAUCUGAACUGUUUGTT |
| Si-NC | UUC UCC GAA CGU GUC ACG UTT | ACG UGA CAC GUU CGG AGA ATT |
| QPCR-CTTN | GCTTTGAGTATCAAGGCAAAACG | CCAAGGGCACATTTGTCTTGT |
| QPCR-GAPDH  QPCR-HBX | ACAGCCTCAAGATCATCAGC GCTCTAGAgccaccaccatgGCTG  CTAGGCTGTGCTGC | GGTCATGAGTCCTTCCACGAT  GCTCTAGATTAGGCAGAGGTGAAAAAGTTG |

**T****able S2 Newly identified HBx-interacting proteins**

| Accession | Description | Score | Coverage | Unique Peptides | PSMs | MW[KDa] | Calc.PI |
| --- | --- | --- | --- | --- | --- | --- | --- |
| Q14247 | Src substrate GN=CTTN | 65.66 | 32.73 | 18 | 33 | 61.5 | 5.4 |
| H7C5W5 | Peripherin(Fragment) GN=PRPH | 5.25 | 5.50 | 1 | 2 | 22.9 | 5.29 |
| P62937 | Peptidyl-prolyl cis-trans isomerase A GN=PPIA | 2.39 | 6.67 | 1 | 1 | 18.0 | 7.81 |
| Q5SYE7 | NHS-like protein 1 GN=NHSL1 | 2.01 | 1.12 | 1 | 1 | 170.6 | 6.96 |
| Q8N1N4 | Keratin, typeⅡcytoskeletal 78 GN=KRT78 | 2.74 | 2.31 | 1 | 1 | 56.8 | 6.02 |
| H0YIN9 | Keratin, typeⅡcytoskeletal 5(Fragment) GN=KRT5 | 2.06 | 4.59 | 1 | 1 | 22.0 | 5.16 |
| A0A140T9X6 | HLA classⅠhistocompatibility antigen, alpha chain F (Fragment) GN=HLA-F | 2.11 | 5.16 | 1 | 1 | 23.9 | 5.02 |
| H0YFX9 | Histone H2A(Fragment) GN=H2AFJ | 3.61 | 11.96 | 1 | 1 | 10.0 | 5.70 |
| P02042 | Hemoglobin subunit delta HN=HBD | 19.20 | 34.69 | 1 | 11 | 16.0 | 8.05 |
| P19876 | C-X-C motif chemokine 3 GN=CXCL3 | 1.62 | 11.21 | 1 | 1 | 11.3 | 10.37 |
| P21964 | Catechol O-methyltransferase GN=COMT | 2.61 | 4.43 | 1 | 1 | 30.0 | 5.47 |
| P63267 | Actin, gamma-enteric smooth muscle GN=ACTG2 | 24.48 | 8.51 | 1 | 10 | 41.8 | 5.48 |
| P47914 | 60S ribosomal protein L29 GN=RPL29 | 2.60 | 9.43 | 1 | 1 | 17.7 | 11.66 |
| H0YDD8 | 60S acidic ribosomal protein P2 (Fragment) GN=RPLP2 | 2.98 | 13.04 | 1 | 1 | 9.1 | 4.46 |
| I3L404 | 40S ribosomal protein S2 (Fragment) GN=RPS2 | 2.64 | 7.10 | 1 | 1 | 16.6 | 10.07 |

**Table S3 Human proteins interacting with CTTN**

| Species | Gene name | UniProtKB/Swiss-Prot |
| --- | --- | --- |
| Homo sapiens | APEH | P13798 |
| Homo sapiens | ATP6V1C1 | P21283 |
| Homo sapiens | FLNA | P21333 |
| Homo sapiens | NCF1 | P14598 |
| Homo sapiens | LMNA | P02545 |
| Homo sapiens | ME1 | P48163 |
| Homo sapiens | RDX | P35241 |
| Homo sapiens | RPA3 | P35244 |
| Homo sapiens | SYNPO | Q8N3V7 |
| Homo sapiens | IQGAP1 | P46940 |
| Homo sapiens | CORO1B | Q9BR76 |
| Homo sapiens | TINF2 | Q9BSI4 |
| Homo sapiens | KIFC3 | Q9BVG8 |
| Homo sapiens | SART3 | Q15020 |
| Homo sapiens | RAD21 | O60216 |
| Homo sapiens | EGLN3 | Q9H6Z9 |
| Homo sapiens | PPP1CB | P62140 |
| Homo sapiens | WIPF1 | O43516 |
| Homo sapiens | NONO | Q15233 |
| Homo sapiens | SYNCRIP | O60506 |
| Homo sapiens | SDC3 | O75056 |
| Homo sapiens | OBSL1 | O75147 |
| Homo sapiens | NPHP4 | O75161 |
| Homo sapiens | CBLL1 | Q75N03 |
| Homo sapiens | NOP9 | Q86U38 |
| Homo sapiens | ABI1 | Q8IZP0 |
| Homo sapiens | MYO19 | Q96H55 |
| Homo sapiens | ACD | Q96AP0 |
| Homo sapiens | VTA1 | Q9NP79 |
| Homo sapiens | HDAC6 | Q9UBN7 |
| Homo sapiens | PAN2 | Q504Q3 |
| Homo sapiens | YES1 | P07947 |
| Homo sapiens | ARHGAP17 | Q68EM7 |
| Homo sapiens | PTPN1 | P18031 |
| Homo sapiens | ELAVL1 | Q15717 |
| Homo sapiens | GSPT2 | Q8IYD1 |
| Homo sapiens | ACTG1 | P63261 |
| Homo sapiens | PAK2 | Q13177 |
| Homo sapiens | PTK2 | Q05397 |
| Homo sapiens | MYO1E | Q12965 |
| Homo sapiens | EP300 | Q09472 |
| Homo sapiens | CHD3 | Q12873 |
| Homo sapiens | NEDD9 | Q14511 |
| Homo sapiens | SH3PXD2A | Q5TCZ1 |
| Homo sapiens | MYLK | Q15746 |
| Homo sapiens | CRB2 | Q5IJ48 |
| Homo sapiens | SUCLG2 | Q96I99 |
| Homo sapiens | EXOC8 | Q8IYI6 |
| Homo sapiens | COPS7A | Q9UBW8 |
| Homo sapiens | SHANK2 | Q9UPX8 |
| Homo sapiens | BTRC | Q9Y297 |
| Homo sapiens | CD2AP | Q9Y5K6 |
| Homo sapiens | DBN1 | Q16643 |
| Homo sapiens | PDK2 | Q15119 |
| Homo sapiens | TNK2 | Q07912 |
| Homo sapiens | CUL3 | Q13618 |
| Homo sapiens | SARS | P49591 |
| Homo sapiens | CTNND1 | O60716 |
| Homo sapiens | WASL | O00401 |
| Homo sapiens | PXN | P49023 |
| Homo sapiens | ARPC3 | O15145 |
| Homo sapiens | ARPC2 | O15144 |
| Homo sapiens | UGDH | O60701 |
| Homo sapiens | MTSS1 | O43312 |
| Homo sapiens | KCNA2 | P16389 |
| Homo sapiens | HEXIM1 | O94992 |
| Homo sapiens | SNX2 | O60749 |
| Homo sapiens | USO1 | O60763 |
| Homo sapiens | GSPT1 | P15170 |
| Homo sapiens | THY1 | P04216 |
| Homo sapiens | UBL4A | P11441 |
| Homo sapiens | KCNA3 | P22001 |
| Homo sapiens | YWHAB | P31946 |
| Homo sapiens | SNX1 | Q13596 |
| Homo sapiens | ARPC4 | P59998 |
| Homo sapiens | PLAUR | Q03405 |
| Homo sapiens | DNM2 | P50570 |
| Homo sapiens | CAPZA2 | P47755 |
| Homo sapiens | SMURF1 | Q9HCE7 |
| Homo sapiens | SDHAF2 | Q9NX18 |
| Homo sapiens | STAT1 | P42224 |
| Homo sapiens | MYO5C | Q9NQX4 |
| Homo sapiens | MYO18A | Q92614 |
| Homo sapiens | SPRR2A | P35326 |
| Homo sapiens | GARS | P41250 |
| Homo sapiens | ESR2 | Q92731 |
| Homo sapiens | MCM7 | P33993 |
| Homo sapiens | MLH1 | P40692 |
| Homo sapiens | MYH9 | P35579 |
| Homo sapiens | CTNNA1 | P35221 |
| Homo sapiens | NPM1 | P06748 |
| Homo sapiens | NTRK1 | P04629 |
| Homo sapiens | ATG7 | O95352 |
| Homo sapiens | SSB | P05455 |
| Homo sapiens | SH3BP2 | P78314 |
| Homo sapiens | CASP3 | P42574 |
| Homo sapiens | ARRB1 | P49407 |
| Homo sapiens | SLX4 | Q8IY92 |
| Homo sapiens | EGFR | P00533 |
| Homo sapiens | RPL9P9 | P32969 |
| Homo sapiens | TRIM15 | Q9C019 |
| Homo sapiens | NHSL2 | Q5HYW2 |
| Homo sapiens | ATF2 | P15336 |
| Homo sapiens | SIRT1 | Q96EB6 |
| Homo sapiens | FER | P16591 |
| Homo sapiens | SNW1 | Q13573 |
| Homo sapiens | SYK | P43405 |
| Homo sapiens | SHMT1 | P34896 |
| Homo sapiens | SHMT2 | P34897 |
| Homo sapiens | GRB2 | P62993 |
| Homo sapiens | CDC37 | Q16543 |
| Homo sapiens | ARRB2 | P32121 |
| Homo sapiens | ASAP1 | Q9ULH1 |
| Homo sapiens | DNM1 | Q05193 |
| Homo sapiens | WASH1 | A8K0Z3 |
| Homo sapiens | EWSR1 | Q01844 |
| Homo sapiens | RPA1 | P27694 |
| Homo sapiens | ACTR3 | P61158 |
| Homo sapiens | ACTR2 | P61160 |
| Homo sapiens | PAK1 | Q13153 |
| Homo sapiens | RPA2 | P15927 |
| Homo sapiens | CDH1 | P12830 |
| Homo sapiens | IDE | P14735 |
